# Polymorphism Pro64His within galectin-3 has functional consequences at proteome level in thyroid cells

**DOI:** 10.3389/fgene.2024.1380495

**Published:** 2024-06-12

**Authors:** Roberto Silvestri, Lorenzo Zallocco, Alda Corrado, Maurizio Ronci, Romina Aceto, Benedetta Ricci, Monica Cipollini, Irene Dell’Anno, Chiara De Simone, Giuseppina De Marco, Eleonora Ferrarini, Daniela Beghelli, Maria Rosa Mazzoni, Antonio Lucacchini, Federica Gemignani, Laura Giusti, Stefano Landi

**Affiliations:** ^1^ Department of Biology, Genetic Unit, University of Pisa, Pisa, Italy; ^2^ Department of Translational Research and New Technologies in Medicine and Surgery, University of Pisa, Pisa, Italy; ^3^ Department of Medical, Oral and Biotechnological Sciences, University “G.D’Annunzio” of Chieti-Pescara, Chieti, Italy; ^4^ COIIM, Interuniversitary Consortium for Engineering and Medicine, Campobasso, Italy; ^5^ Department of Clinical and Experimental Medicine, University of Pisa, Pisa, Italy; ^6^ School of Biosciences and Veterinary Medicine, Via Gentile III da Varano, University of Camerino, Camerino, Italy; ^7^ Department of Pharmacy, University of Pisa, Pisa, Italy; ^8^ School of Pharmacy, University of Camerino, Camerino, Italy

**Keywords:** galectin-3 (gal3), functional polymorphism rs4644, differentiated thyroid carcinoma (DTC), CRISPR/Cas9, proteome, papillary thyroid carcinoma (PTC)

## Abstract

**Introduction:**

The single nucleotide polymorphism (SNP) rs4644 at codon 64 of galectin-3 (gal-3, gene name: *LGALS3*), specifying the variant proline (P64) to histidine (H64), is known to affect the protein’s functions and has been associated with the risk of several types of cancer, including differentiated thyroid carcinoma (DTC).

**Materials and methods:**

To deepen our understanding of the biological effects of this SNP, we analyzed the proteome of two isogenic cell lines (NC-P64 vs. NA-H64) derived from the immortalized non-malignant thyrocyte cell line Nthy-Ori, generated through the CRISPR-Cas9 technique to differ by rs4644 genotype. We compared the proteome of these cells to detect differentially expressed proteins and studied their proteome in relation to their transcriptome.

**Results:**

Firstly, we found, consistently with previous studies, that gal-3-H64 could be detected as a monomer, homodimer, and heterodimer composed of one cleaved and one uncleaved monomer, whereas gal-3-P64 could be found only as a monomer or uncleaved homodimer. Moreover, results indicate that rs4644 influences the expression of several proteins, predominantly upregulated in NA-H64 cells. Overall, the differential protein expression could be attributed to the altered mRNA expression, suggesting that rs4644 shapes the function of gal-3 as a transcriptional co-regulator. However, this SNP also appeared to affect post-transcriptional regulatory mechanisms for proteins whose expression was oppositely regulated compared to mRNA expression. It is conceivable that the rs4644-dependent activities of gal-3 could be ascribed to the different modalities of self-dimerization.

**Conclusion:**

Our study provided further evidence that rs4644 could affect the gal-3 functions through several routes, which could be at the base of differential susceptibility to diseases, as reported in case-control association studies.

## 1 Introduction

Galectin-3 (gal-3) is a lectin that participates in many cellular functions, ranging from cell growth, differentiation and proliferation to inflammation, cell-cell and cell-matrix interactions. Similar to other lectins, gal-3 possesses a carbohydrate recognition domain (CRD) that confers to these proteins their typical ability to crosslink glycosylated ligands. However, among the 15 known galectins, gal-3 is the only one with a disordered N-terminal domain that seems to promote its oligomerization and confer unique functions to this lectin. As such, gal-3 can be involved in the onset of many human diseases, including cancer, fibrosis, and inflammation (Sciacchitano et al., 2018). The link between gal-3 and cancer has been mainly studied in thyroid carcinoma (TC). An increased expression of gal-3 has been observed in malignant thyroid tissues, and this correlated with poor prognosis, cancer progression, and metastasis (Paron et al., 2003; Giusti et al., 2008; Song et al., 2012; Li et al., 2019; Wang et al., 2019). Moreover, increased gal-3 serum levels were measured in TC patients compared to controls. For this reason, serum gal-3 was proposed and used as a biomarker for the prognosis of papillary thyroid carcinoma (PTC) (Giusti et al., 2008). More evidence also showed that the over-expression of gal-3 is not the consequence but rather one of the drivers of the malignant transformation of the thyroid (Elad-Sfadia et al., 2004; Levy et al., 2010). Interestingly, single nucleotide polymorphisms (SNPs) within the gal-3 coding gene (*LGALS3*) have been associated with cancer risk, adding further evidence to the studies suggesting the carcinogenic roles of this protein. In particular, the rs4644, encoding for a proline to histidine substitution at codon 64 of gal-3 [P64 > H64], was associated with variable risks of breast (Balan et al., 2008), prostate, cervical (Meyer et al., 2013; Fang et al., 2017), and differentiated thyroid carcinoma (DTC) (de Boer et al., 2012; Song et al., 2014). Moreover, other studies also reported associations with other types of cancer for SNPs in strong linkage disequilibrium with rs4644, such as the case for rs4652 and the risk of gastric carcinoma (Shi et al., 2017) or the molecular characteristics of colorectal cancer (Korkmaz et al., 2016) suggesting more mechanisms involving gal-3 in tumorigenesis.

Given its high conservation among mammals and vertebrates, rs4644 was predicted to change gal-3 biological activity, prompting *in vitro* and *in vivo* studies. Thus, it was found that in gal-3-deprived breast cancer cells, the forced expression of H64 or P64 produced two forms of gal-3 not equally cleaved by matrix metalloproteinases (MMPs) −2 and −9. Moreover, in the same study, H64 and P64 conferred different abilities of chemotaxis, chemo-invasion, angiogenesis, and sensitivity to death receptor-mediated apoptosis (TRAIL-pathway). P64 variant-expressing cells, when xenografted in nude mice, have also shown reduced angiogenesis and tumor progression (Nangia-Makker et al., 2007; Nangia-Makker et al., 2010; Mazurek et al., 2011).

Furthermore, in a large case-control association study, we found that the variant H64 allele of rs4644 was associated with a reduced risk of DTC compared to P64 (Corrado et al., 2021), and the association was also found in the repository offered by the public database “UK BioBank” (Bycroft et al., 2018) following the analysis with Phenoscanner (Kamat et al., 2019) and Open Targets Genetics (Ghoussaini et al., 2021). Thus, we recently evaluated the hypothesis that P64 and H64 gal-3 variants are not functionally equivalent in thyroid cells. By using the CRISPR/Cas9 technique on the non-malignant thyroid follicular epithelial cells Nthy-Ori, we generated two isogenic cell lines that were homozygous either for the CC/gal-3P64 (defined as NC-P64 cells) or the AA/gal-3H64 (NA-H64) genotype at the rs4644 (Corrado et al., 2023). Since nuclear gal-3 is known to bind and cooperate with transcription factors (Guazzi et al., 1990; Paron et al., 2003; Takenaka et al., 2003; Gilbert-Sirieix et al., 2011), in a previous study, we evaluated the transcriptome of these cells as well as that of the original Nthy-Ori (heterozygous for the rs4644) (Corrado et al., 2021).

This previous study showed that the mRNA expression of several genes was dependent on the *LGALS3* genotype (Corrado et al., 2021), confirming the gal-3 involvement in gene expression and suggesting a role for the rs4644 in modulating this activity. However, a detailed molecular analysis of protein expression changes in these cells was still missing. In the present work, to assess the effect that the rs4644 may exert on protein expression, we evaluate and compare the proteomic profiles of the engineered NC-P64 and NA-H64 cells. To provide a mechanistic explanation for the observed results, we also put these data in relation to those from our aforementioned transcriptomic study.

## 2 Results

### 2.1 Gal-3 protein expression in NC-P64 and in NA-H64 cells

**FIGURE 1:**
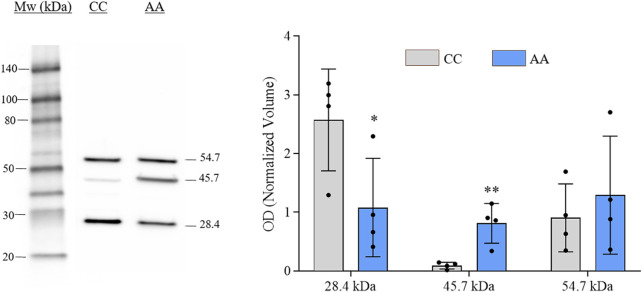
Image showing the gal-3 immunoblot analysis conducted on NA-H64 (AA) and NC-P64 (CC) protein extracts. The bar graph shows the mean ± SEM of the normalized OD values. Ruthenium staining was used as a protein-loading control. Statistical differences in the immunoreactive bands of different samples were calculated using a non-parametric unpaired t-test. *p < 0.05, **p < 0.01.

The verification of gal-3 knock-in in engineered cells was performed by western blot (WB) analysis. Representative immunoblot and histograms of the OD values (mean ± SEM) of each immunoreactive band for NA-H64 and NC-P64 samples are shown in Figure 1. Three immunoreactive bands were detected in both samples at approximately 28, 46, and 55 kDa. The ∼28 kDa and ∼55 kDa bands represent the monomeric and dimeric form of gal-3, respectively. The ∼46 kDa band is compatible with the notion that the AA form (H64) can be cleaved by cellular MMPs acting on the restriction site at Ala62-Tyr63, eliciting a truncated gal-3 lacking ∼9 KDa (Nangia-Makker et al., 2010). The blot is consistent with previous findings showing that the cleaved form is present almost exclusively in NA-H64 cells. Therefore, the ∼46 KDa is likely the result of a heterodimerization of an uncleaved monomer with a cleaved one. However, when summing the band intensities, no significant changes in total gal-3 expression were observed between NA-H64 and NC-P64 samples.

### 2.2 Differential proteomics analysis

**FIGURE 2:**
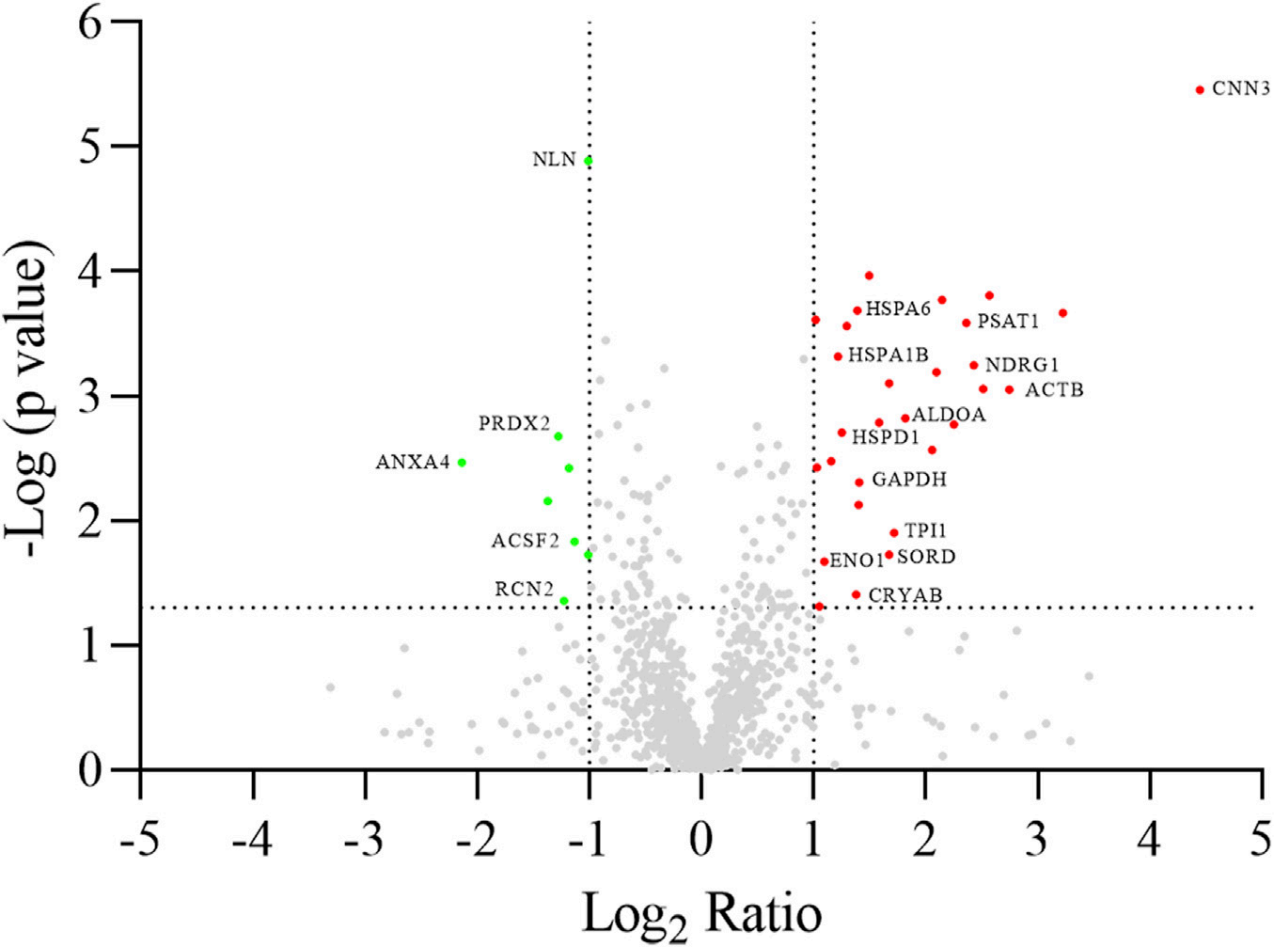
Volcano plot of log2 ratio (x-axis) against log *p*-value (y-axis) of all quantified proteins showing both the significantly increased (red) and significantly decreased (green) hits. Gene names are reported according to Table 1. The dotted lines indicate the thresholds of significance (*p*-value <0.05) and fold change (fold change ≥2), respectively.

A differential analysis between NC-H64 and NA-P64 cells was performed using two-dimensional electrophoresis (2-DE). About 1,300 protein spots were detected on gels from different samples (Supplementary Figure S1). The computational comparison of the images identified 35 protein spots differentially expressed in NA-H64 compared to NC-P64 cells. To identify the proteins potentially correlated with rs4644 polymorphism, differentially expressed spots were cut from gels, trypsin digested, and analyzed by nano-LC ESI MS/MS. A volcano plot of spot distribution obtained by the comparison is shown in Figure 2, whereas the list of identified proteins, with their molecular weight (MW), isoelectric point (pI), coverage values of MS/MS ratios, and their relative *p*-values are shown in Table 1. Among the differentially expressed proteins, CNN3 showed the largest change with a more than 20-fold increased spot intensity in NA-H64 compared to NC-P64 protein extracts. In addition to the main spot (pI 6.09), an acidic CNN3 spot was observed (Table 1). More phosphorylation at different serine and threonine residues, which play a crucial role in signaling activation, are described for CNN3 and other isoforms (CNN1, CNN2) (Supplementary Figure S2) (Shibukawa et al., 2013; Maddala et al., 2020). Moreover, NA-H64 cells showed a > 2-fold increased expression of several other proteins such as ACTB, ADH5, ALDOA, CRYAB, DLST, ENO1, GAPDH, HNRNPC, HNRNPD, HSPA1A, HSPA1B, HSPA6, HSPA8, HSPB1, HSPD1, MAP2K2, NDRG1, PACSIN2, PAPSS2, PSAT1, RELA, SOD2, SORD, and TPI1, and a decreased expression of PPM1G, NLN, ACSF2, PEPD, RCN2, ANXA4, NPM1, PRDX2, and CMPK1. WB analysis confirmed a statistically significant difference between the cell lines for two proteins (i.e., TPI1 and ALDOA), which were used as representative examples (Supplementary Figure S3).

**TABLE 1:** List of differentially expressed proteins identified by LC/MS/MS spectrometry.

*PROTEOMICS*									*TRANSCRIPTOMICS*		
Spot n°	ID^a^	Gene	Cov^b^%	Pept Uniq	MW^c^	pI^d^	*p*-value	Ratio (AA/CC)	FPKM value (AA)	FPKM value (CC)	Ratio (AA/CC)
*PROTEINS WITH SIGNIFICANTLY HIGHER EXPRESSION IN AA-GENETIC BACKGROUND*											
552	P11142	*HSPA8*	17	6	70,898	5.37	2.44E-04	2.0	1.58	1.10	1.4
623	P17066	*HSPA6*	32	12	71,028	5.81	2.06E-04	2.6	94.86	0.03	3,259.5
632	P10809	*HSPD1*	4	4	61,055	5.24	0.002	2.4			
656	P0DMV8	*HSPA1A*	57	46	70,052	5.47	4.81E-04	2.3			
656	P0DMV9	*HSPA1B*	57	46	70,052	5.47	4.81E-04	2.3	5.16	0.00	>>1
656	Q04206	*RELA*	13	7	59,910	5.46	4.81E-04	2.3	197.59	98.20	2.0
667	Q9UNF0	*PACSIN2*	4	2	51,353	5.26	2.74E-04	2.4	36.88	31.87	1.2
683	O95340	*PAPSS2*	6	3	69,501	8.18	0.003	2.2	27.34	9.09	3.0
769	P06733	*ENO1*	18	4	36,928	5.93	0.021	2.1	1,014.98	726.00	1.4
935	P36957	*DLST*	10	4	48,755	5.90	1.08E-04	2.8	55.61	58.98	0.94
1,003	Q92597	*NDRG1*	12	2	42,835	5.49	5.63E-04	5.4			
1,004	P60709	*ACTB*	10	3	41,737	5.29	8.74E-04	5.7	1,171.41	1823.32	0.6
1,010	P36507	*MAP2K2*	12	3	44,424	6.12	1.55E-04	5.9	74.65	43.35	1.7
1,085	P04075	*ALDOA*	28	9	39,420	8.39	0.002	3.5	448.21	156.31	2.9
1,131	Q9Y617	*PSAT1*	20	7	40,423	7.56	2.59E-04	5.1	196.95	564.65	0.3
1,131	P11766	*ADH5*	5	2	39,724	7.45	2.59E-04	5.1	84.44	64.88	1.3
1,134	Q00796	*SORD*	20	5	38,325	8.25	0.019	3.2	9.34	8.39	1.1
1,134	Q14103	*HNRNPD*	9	2	36,272	8.14	0.019	3.2	77.72	102.13	0.8
1,138	P07910	*HNRNPC*	7	2	32,338	4.94	0.002	4.8	43.04	54.07	0.8
1,141	Q15417	*CNN3*	25	5	31,380	6.09	0.007	2.6			
1,143	Q15417	*CNN3*	7	2	31,380	6.09	3.47E-06	21.8	136.85	123.53	1.1
1,187	P04406	*GAPDH*	9	2	36,053	8.58	0.005	2.7	2,647.24	1,552.99	1.7
1,506	P04792	*HSPB1*	49	11	22,783	5.98	0.002	3.0	978.75	101.71	9.6
1,543	P60174	*TPI1*	31	6	26,669	6.45	0.013	3.3	564.22	342.42	1.6
1,655	P04179	*SOD2*	26	4	19,730	7.81	7.88E-04	3.2	27.06	12.28	2.2
1710	P02511	*CRYAB*	26	4	20,159	6.76	0.049	2.0	141.78	15.21	9.3
*PROTEINS WITH SIGNIFICANTLY LOWER EXPRESSION IN AA-GENETIC BACKGROUND*											
551	O15355	*PPM1G*	44	18	59,272	4.27	0.007	0.4	116.41	143.97	0.8
562	Q9BYT8	*NLN*	17	10	80,652	5.77	1.29E-05	0.5	15.94	11.73	1.4
723	Q96CM8	*ACSF2*	19	10	68,125	6.40	0.015	0.5	47.19	25.16	1.9
828	P12955	*PEPD*	15	7	54,548	5.64	0.019	0.5	15.45	18.61	0.8
907	Q14257	*RCN2*	8	2	36,876	4.22	0.044	0.4	19.61	22.31	0.88
1,179	P09525	*ANXA4*	18	5	35,883	5.84	0.003	0.2	11.69	21.68	0.5
1,222	P06748	*NPM1*	28	4	32,575	4.64	0.004	0.4	223.94	383.78	0.6
1,650	P32119	*PRDX2*	36	6	21,892	5.67	0.002	0.4	32.59	93.62	0.3
1,650	P30085	*CMPK1*	19	3	22,222	5.44	0.002	0.4	39.86	63.84	0.6

^a^ ID: Uniprot accession number.

^b^ Cov: coverage.

^c^ MW: molecular weight.

^d^ pI: isoelectric point.

FPKM: “Fragments Per Kilobase of exon Per Milion mapped reads”.

### 2.3 Protein and mRNA expression analysis in NC-P64 and NA-H64 cells

As reported in Table 1, we compared the results of the differentially expressed proteins with their transcription profiles previously measured in NA-H64 and NC-P64 cells. This comparison was made possible thanks to our previous study on the same cells (Corrado et al., 2021), showing the effect of rs4644 at the nuclear level, gal-3 being a co-regulator of gene transcription. Among the 31 differentially expressed proteins for which the mRNA expression levels were available, most showed a concordance between the AA/CC protein expression ratio and the AA/CC mRNA expression ratio, i.e., the direction of the fold change was the same when compared to the one obtained from mRNA data. In particular, 17 proteins with an AA/CC ratio>1.1 also had a ratio >1.1 at the mRNA level. Moreover, 7 proteins with a lower expression in the AA background (i.e., AA/CC < 0.9) also had a ratio<0.9 at the mRNA level. Thus, 24 (77%) had the same expression trend at both the mRNA and the protein level. Despite a not varied mRNA expression, one protein (DLST) showed about 2.8-fold increased expression in NA-H64 compared to NC-P64 cells. Finally, six proteins showed an opposite trend. Indeed, NA-H64 cells showed, when compared to NC-P64 cells, an increased expression of ACTB, PSAT1, HNRNPD, and HNRNPC despite a reduced mRNA expression, while NLN and ACSF2 had a reduced expression despite an increased mRNA expression.

### 2.4 Pathway analysis

**FIGURE 3:**
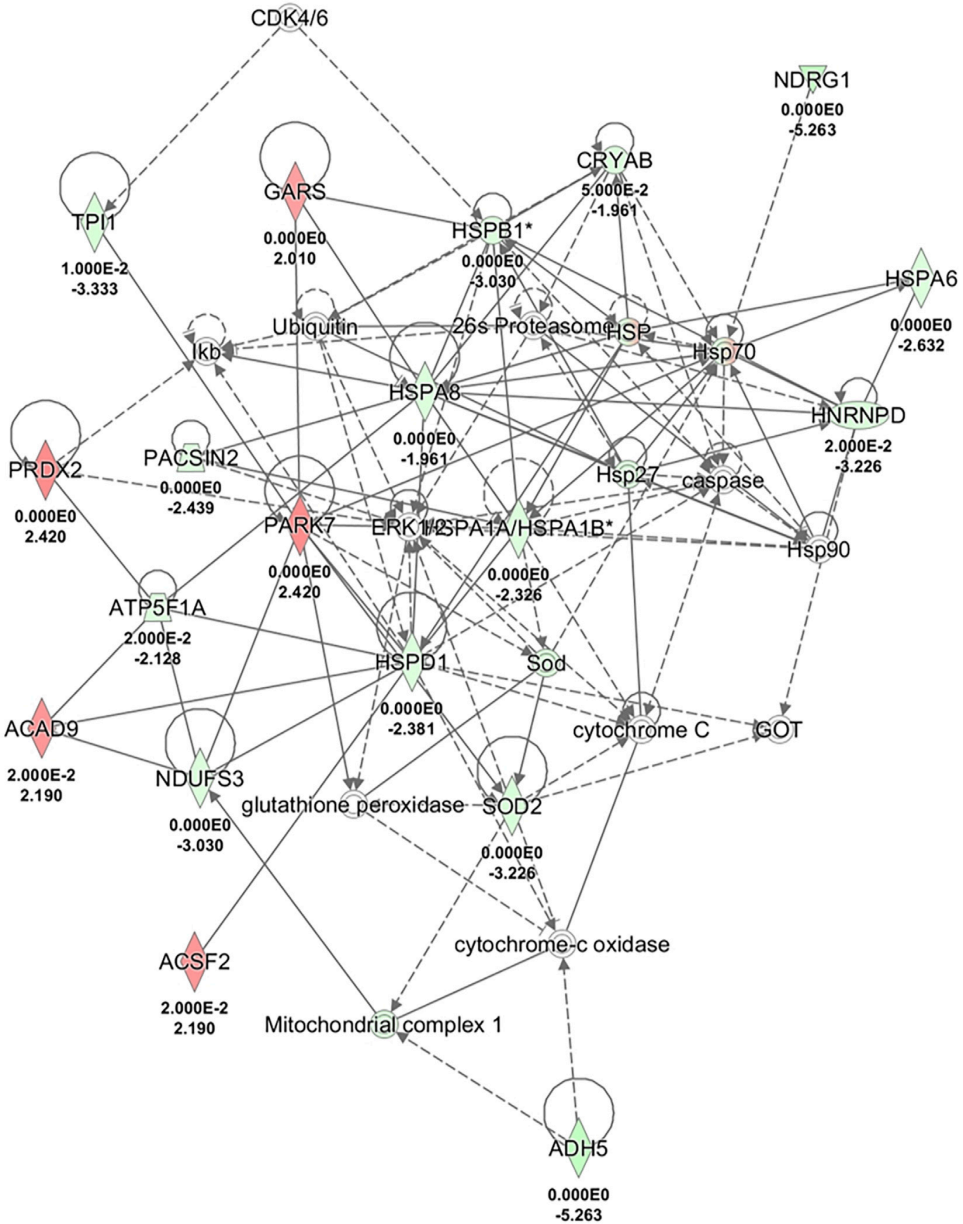
Network analysis of differentially expressed proteins in the comparison in AA with comparison to CC cells using IPA software. The network shows proteins interactions in the context of “Post-translational modification, protein folding, endocrine system disorder”, along with corresponding protein-to-protein direct (solid line) or indirect (dashed line) interactions based on published literature information.

All identified differentially expressed proteins were included in bioinformatics analyses to highlight possible alterations in molecular pathways. When using Ingenuity Pathway Analysis, 16 of the 35 input proteins were associated with the “post-translational modification, protein folding, endocrine system disorder” with a *p*-value of 0.025 and a score of 40 (Figure 3). When Reactome was used, the abundance of heat shock proteins found in NA-H64 cells was tagged as highly statistically significant, spotting the activation of HSF1 transcription factor as the main differentially activated pathway in the AA genetic background (*p* < 10^−15^).

## 3 Discussion

In the present work, we added evidence that the common gal-3P64 variant is not functionally equivalent to the alternative gal-3H64 variant. According to public databases, the proline residue at this codon is highly conserved throughout evolution, and the histidine variant is present only in humans. Unlike other galectins, gal-3 can assemble into oligomers whose valency can affect its functions (Nangia-Makker et al., 2018). In particular, dimerization is thought to occur through the reciprocal interaction between a disordered N-terminal domain (NTD) and the carbohydrate-recognition domain (Lin et al., 2017). It has also been shown that the NTD of gal-3H64, but not that of gal-3P64, can be cleaved by the proteolytic activities of MMP2 and MMP9 (Ochieng et al., 1994; Nangia-Makker et al., 2010).

In agreement with this notion, we found that the NA-H64 cells can express a heterodimer composed of a cleaved subunit and an uncleaved one, while, in the NC-P64 cells, only the homodimer of two uncleaved monomers and the monomer were detectable. This fact is strongly indicative that the extent of dimerization is genotype-dependent, and it could explain part of the differential biological effects of the two alleles (Dumic et al., 2006; Chiu et al., 2020; Farhadi et al., 2021).

In addition, our work clearly showed that the proteomic profile in the isogenic cell lines NA-H64 and NC-P64 is different, with 25 proteins overexpressed and 9 downregulated in NA-H64 compared with NC-P64 cells. For 31 of these proteins, we had previous data on the mRNA expression levels. Interestingly, when we compared the protein and the mRNA expression, we found the same trend in 24 out of 31 cases (77%). These results confirmed our previous findings on the transcriptome of these cells (Corrado et al., 2021) and, in turn, can be explained by an rs4644-dependent transcriptional activity of gal-3. In fact, gal-3 is known to function in the nucleus as a co-regulator of many transcription factors, such as TTF-1 and TCF-4 (Guazzi et al., 1990; Paron et al., 2003; Takenaka et al., 2003; Gilbert-Sirieix et al., 2011). Therefore, overall, we can conclude that the rs4644-AA genotype, when compared to the common CC genotype, is associated with or causes deregulation of the transcription of 24 mRNAs, and this altered transcription also causes an altered expression of the encoded proteins.

Among the 24 mRNAs, 21 were upregulated in the AA genetic background, suggesting an enhanced transcriptional nuclear activity of gal-3 associated with this genotype. Interestingly, when an increased nuclear expression of gal-3 was induced in engineered prostate cancer cells, inhibition of malignant behaviors was observed (Califice et al., 2004). *In vitro*, these cells exhibited reduced Matrigel invasion and limited anchorage-independent growth, while, *in vivo*, they showed reduced tumor growth and angiogenesis and increased inducible apoptosis (Califice et al., 2004). Thus, we could speculate that the reduced risk of DTC associated with the rs4644-A allele (Corrado et al., 2021) may be ascribed (at least partially) to an increased nuclear activity of gal-3H64.

Concerning ACTB, DLST, PSAT1, HNRNPD, and HNRNPC proteins (higher protein expression in NA-H64 than in NC-P64 cells with reduced or unaltered mRNA expression), the effect of the polymorphism is less obvious. However, it has been clearly shown that, in the cytosol, gal-3 binds the heterogeneous nuclear ribonucleoprotein hnRNP-L that, in turn, binds mRNAs (Coppin et al., 2017). This complex is pivotal in regulating mRNA stability and translation rates (Gu et al., 2020) by binding to CAREs (CA-repeated elements) of mRNAs (Coppin et al., 2017; Venkata Subbaiah et al., 2019). When hnRNP-L binds CAREs within the 5′UTR, the complex works as an ITAF (internal ribosome entry site element trans-acting factor), crucial for enhancing translation initiation (Seo et al., 2017). When hnRNP-L binds CAREs within the 3′UTR, the complex can elicit different effects on the intensity of translation by stabilizing the mRNA or cooperating or competing with translation inhibitors such as the RISC-miRNAs or GAIT complexes (Venkata Subbaiah et al., 2019). Since DLST, HNRNPC, and PSAT1 bear CAREs (Venkata Subbaiah et al., 2019), it could be speculated that the gal-3-hnRNPL complex has a differential activity on mRNAs, depending upon the rs4644 genotype.

For ACTB and HNRNPD, we should evoke hitherto unknown mechanisms of rs4644-dependent post-transcriptional regulation. Similar mechanisms could also affect CNN3 (calponin-3) since this protein showed the largest increase of expression (21.8-fold) in association with the rs4644-AA genotype, which is not explainable by the modest increase of mRNA expression (1.1-fold). Interestingly, CNN3 binds actin, another protein strongly upregulated (5.7-fold) in the NA-H64 cells. CNN3 activity is poorly known, but recent studies have highlighted a role in modulating cells’ motility and contractile ability. Moreover, a role in modifying the Yap/Taz-dependent transcriptional activation was suggested. Thus, CNN3 and/or its phosphorylated form could play a role in cell differentiation, proliferation, and migration via stress fiber formation or cytoskeletal remodeling (Shibukawa et al., 2013; Liu and Jin, 2016; Maddala et al., 2020).

Among the proteins whose low expression was associated with the rs4644-AA genotype, PRDX2 (peroxiredoxin 2) caught our attention. This enzyme is involved in the oxy-reductive pathways of thyrocytes for thyroid hormone production, and it has been previously reported as one of the key upregulated enzymes in thyroid carcinoma (Netea-Maier et al., 2008). Moreover, in a proteomic study of thyroid carcinoma cells (Trojanowicz et al., 2010), it has been shown that this enzyme is strongly downregulated when cells are treated with retinoic acid. This change was also associated with differentiation, reduced proliferation, and lower invasive capacities of the cells. Although it is unknown whether these anti-malignant phenotypes are caused by or simply associated with PRDX2 decrease, it is interesting to note that the genotype associated with a reduced PRDX2 protein level was also associated with a reduced risk of DTC (Corrado et al., 2021).

The change of expression of 34 proteins, once analyzed for potentially altered molecular pathways using Reactome, suggested a link between rs4644 and the level of the heat-shock and stress-related proteins (HSPs), likely driven by a higher activity of HSF-1 transcription factor in NA-H64 than NC-P64 cells. These observations suggest that the NA-H64 cells are more constitutively stimulated as they were in a higher stress state compared to NC-P64 cells. HSPs play a pivotal role in controlling the correct folding of newly synthesized proteins, assuring their functional conformation, and preventing the aggregation of damaged proteins. The involvement of rs4644 in post-translational modification and protein folding was also confirmed using an independent algorithm (Ingenuity Pathway Analysis). Among the stress-related proteins, we noticed NDGR1 was highly expressed in NA-H64 cells. This protein is a tumor suppressor in many cell types (Ghafouri-Fard et al., 2023). It is important for p53-mediated caspase activation/apoptosis and mitotic spindle checkpoint. It protects cells from aberrant mitotic spindle formation, helping to maintain euploidy (Ghafouri-Fard et al., 2023). These data suggest that a slightly higher activation of stress pathways associated with the rs4644-AA genotype protects thyrocytes from a malignant transformation.

In summary, the present study provided further information on the biological effects of the rs4644 polymorphism, shedding new light on possible mechanisms of its association with human susceptibility to cancer.

## 4 Materials and methods

### 4.1 Cell cultures and gene editing

The non-malignant human thyroid cell line Nthy-Ori (Sigma-Aldrich, Saint Louis, MO, United States) was employed for gene editing and proteomic analysis. The cells were grown in medium RPMI 1640 supplemented with 10% fetal bovine serum (FBS) (EuroClone SpA, Milan, Italy). Nthy-Ori cells have the heterozygous *LGALS3* genotype CA at rs4644, allowing their conversion into homozygous genotype (either AA or CC) using the CRISPR/Cas9 gene editing system, as extensively described by Corrado et al. (Corrado et al., 2021).

### 4.2 Protein extraction and two-dimensional electrophoresis (2-DE)

Sub-confluent cells were detached by trypsinization, collected, centrifuged at 500 g for 5 min, then washed with PBS and centrifuged again. The resulting cellular pellets were re-suspended in the rehydration solution (7 M urea, 2 M thiourea, 4% CHAPS, 60 mM DTT), sonicated, and incubated for 1 h at room temperature (RT) with occasional stirring.

The solution was then centrifuged at 17,000 g for 15 min at RT to eliminate insoluble materials. Two-dimensional electrophoresis (2-DE), gel staining, and image acquisition and analysis were carried out as previously described (Ciregia et al., 2013; Lacerenza et al., 2020). The significance of the differences in normalized volume for each spot was calculated using the ANOVA test, and the protein spots that showed *p*-value < 0.05, q value < 0.05, and fold variation ≥2 fold were selected and cut out from the gel for identification by LC-MS/MS.

### 4.3 In-gel digestion and mass spectrometry

The gel pieces were digested and analyzed by LC-MS/MS using a Proxeon EASY-nLCII (Thermo Fisher Scientific, Milan, Italy) chromatographic system coupled to a Maxis HD UHR-TOF (Bruker Daltonics GmbH, Bremen, Germany) mass spectrometer as previously described (Ciregia et al., 2015; Giusti et al., 2018). Raw data were processed with Data Analysis v. 4.2 to apply the lock mass calibration and then loaded in PEAKS Studio v7.5 software (Bioinformatic Solutions Inc., Waterloo, Canada) using the ‘correct precursor only’ option. The mass lists were searched against the NextProt database (downloaded in March 2023 and containing 51,692 entries). Non-specific cleavage was allowed to one end of the peptides, with a maximum of 2 missed cleavages and 2 variable PTMs per peptide. Ten ppm and 0.05 Da were set as the highest error mass tolerances for precursors and fragments, respectively. A −10lgP threshold for PSMs was manually set to 35.

### 4.4 Western blot analysis

Western blot was carried out on cellular samples to evaluate the level of gal-3 expression and validate 2DE and network analysis results. To this end, fructose-bisphosphate aldolase A (ALDOA) and triosephosphate isomerase (TPI1) expression changes were assayed as representative proteins.

For each sample, 20 µg of proteins were mixed with the Laemmli solution and ran in 4%–15% polyacrylamide gels (Mini-PROTEAN^®^ Precast Gels, Bio-Rad) using a mini-Protean Tetracell (Bio-Rad). The proteins were transferred onto nitrocellulose membranes (0.2 µm) using a Trans-Blot Turbo transfer system (Bio-Rad). The following antibodies were used: rabbit anti-gal-3 (sc-20157, Santa Cruz) dilution 1:10,000; goat anti-ALDOA (sc-12059 Santa Cruz) and goat anti-TPI1 (ab-28760 Abcam) dilution 1:5,000. The HRP-goat anti-rabbit (Enzo Life Sciences, Inc., NY, United States) and HRP-goat anti-mouse (PerkinElmer, Inc., MA, United States) secondary antibodies were used at 1:10,000 dilution, whereas the HRP-donkey anti-goat was used at 1:5,000 dilution. Immunoblots were developed using the enhanced chemiluminescence detection system (ECL), and the chemiluminescent images were acquired using LAS4010 (GE Healthcare). The immunoreactive specific bands were quantified using Image Quant-L software. In order to normalize the optical density (OD) of immunoreactive bands, the optical density of total proteins was calculated. After the electroblot, membranes were stained with 1 µM RuBPS.

### 4.5 Network analysis

Proteins differentially expressed by the NA-H64 vs. NC-P64 comparison were functionally analyzed using the Ingenuity Pathway Analysis (IPA, QIAGEN Redwood City, United States, www.qiagen.com/ingenuity, Build version: 321501 M Content version: 21249400) which allows to determine the predominant canonical pathways and interaction network involved (Krämer et al., 2014).

The created networks describe functional relationships among proteins based on known associations in the literature. A confirmatory analysis was carried out using the Reactome Pathway Database, giving the differentially expressed proteins as input (Gillespie et al., 2022).

### 4.6 Statistical analysis

Calculations were performed using GraphPad Prism 8.4.2 (GraphPad Inc., San Diego, CA, United States) and SPSS Statistics 20.0 (SPSS Inc., Chicago, IL, United States). When variables were not normally distributed, differences between NA-H64 and NC-P64 cells were analyzed using the Mann-Whitney U test for non-normal data.

## Data Availability

The original contributions presented in the study are included in the article/Supplementary Material; further inquiries can be directed to the corresponding authors.
