# Supplementary material for: Polymorphism Pro64His within galectin-3 has functional consequences at proteome level in thyroid cells
Source: Front Genet. 2024 Jun 12;15:1380495. doi: 10.3389/fgene.2024.1380495 (PMC11199678; doi:10.3389/fgene.2024.1380495)
Supplement: Supplementary file 1 [file DataSheet1.ZIP › Supplementary_Matherial/Supporting_Information_Captions.docx]

**Supporting information captions**

**Supplementary Figure S1:** 2DE representative image of NTH-ORI cell protein extracts. Proteins were separated in a 3–10 nonlinear gradient. SDS-PAGE was performed using 12% acrylamide. Gels were stained with ruthenium. Gene names of differentially expressed spot were reported.

**Supplementary Figure S2:** Peptide mass spectra of two peptides used for protein identification. The figure depicts the MS/MS spectrum of peptides, LGPSYGLSAEVK and LTLQPVDNSTISLQMGTNK, respectively. The identified y- and b-fragments are indicated in the tables below the spectrum. Three additional peptides identified the CCN3 protein.

**Supplementary Figure S3:** Validation of two differentially expressed proteins, ALDOA and TPI1, in ORI-AA with comparison to ORI-CC cells using WB analysis. Histograms of the normalized OD obtained in ORI-AA and ORI-CC samples are reported. Data are presented as mean ± SEM of three experiments. Representative bands are shown below the histograms. A single immunoreactive band with apparent molecular weight approximately of 44 kDa and 25 kDa was obtained for ALDOA and TPI1, respectively. Statistical analysis was performed using a non-parametric unpaired t test. *p<0.05, **p<0.01.
