# Supplementary figures and images for: Polymorphism Pro64His within galectin-3 has functional consequences at proteome level in thyroid cells
Source: Front Genet. 2024 Jun 12;15:1380495. doi: 10.3389/fgene.2024.1380495 (PMC11199678; doi:10.3389/fgene.2024.1380495)

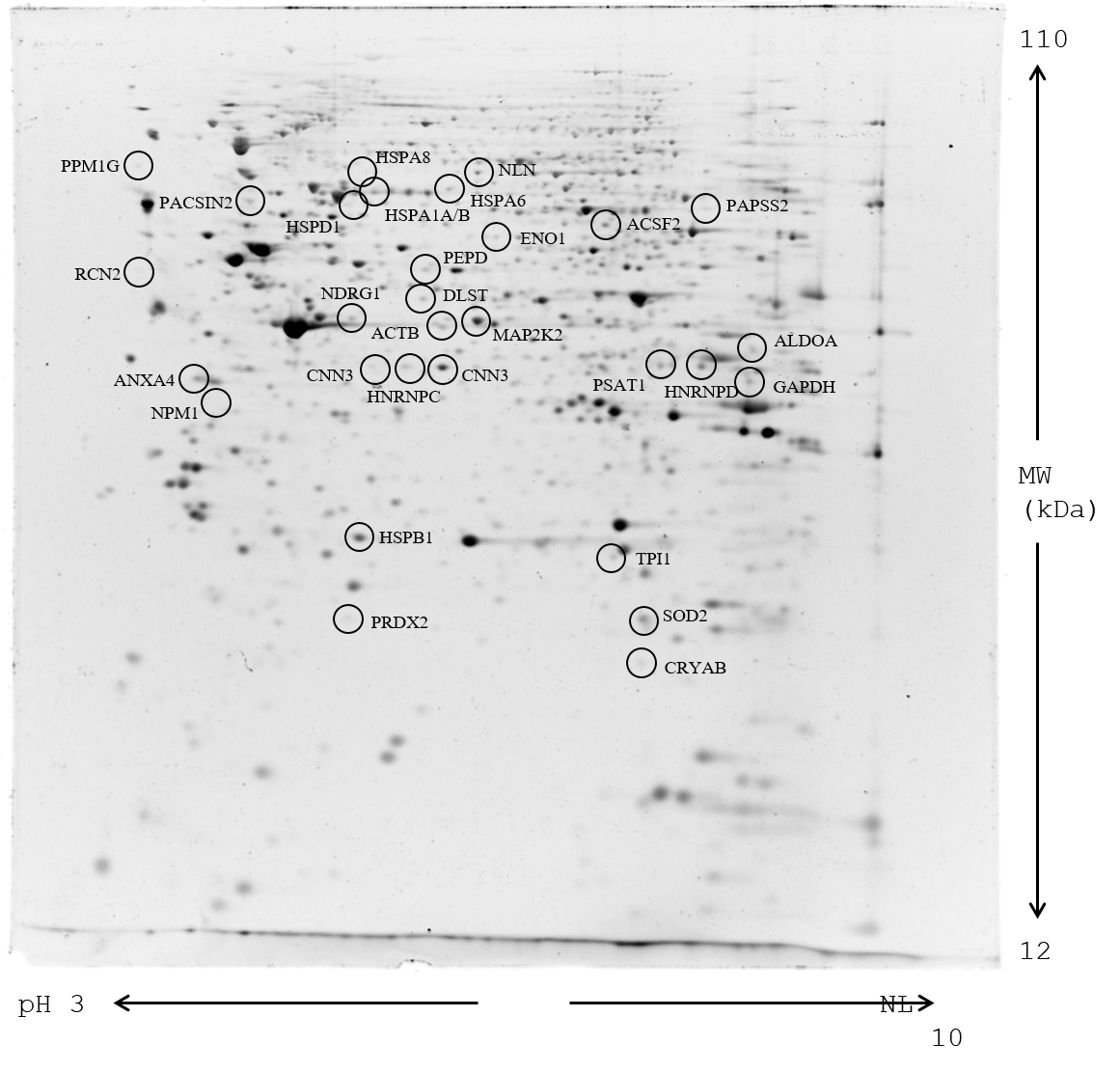

Supplement: Supplementary file 1 [file DataSheet1.ZIP › Supplementary_Matherial/Supplementary_Figure_S1.tif]

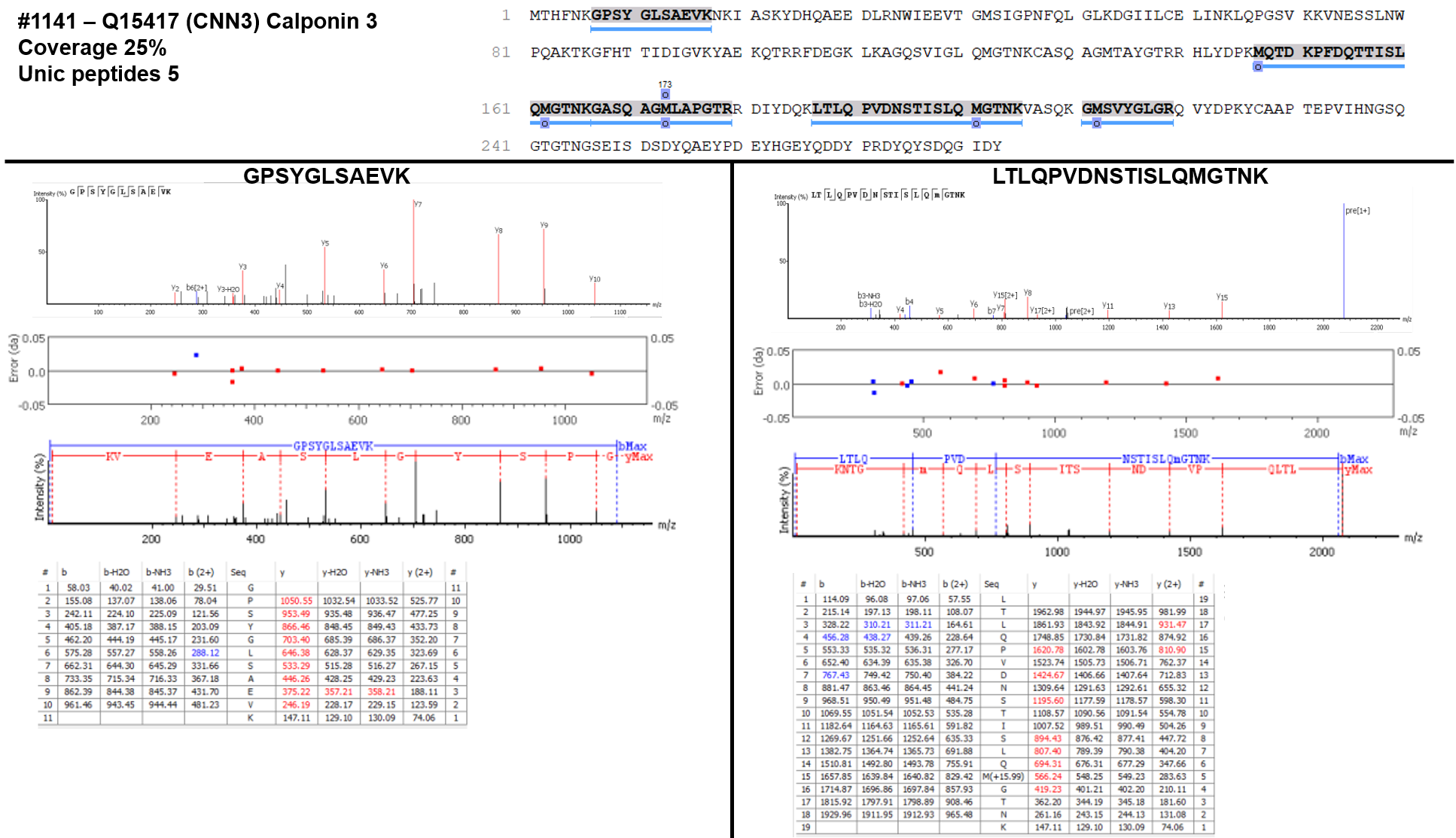

Supplement: Supplementary file 1 [file DataSheet1.ZIP › Supplementary_Matherial/Supplementary_Figure_S2.tif]

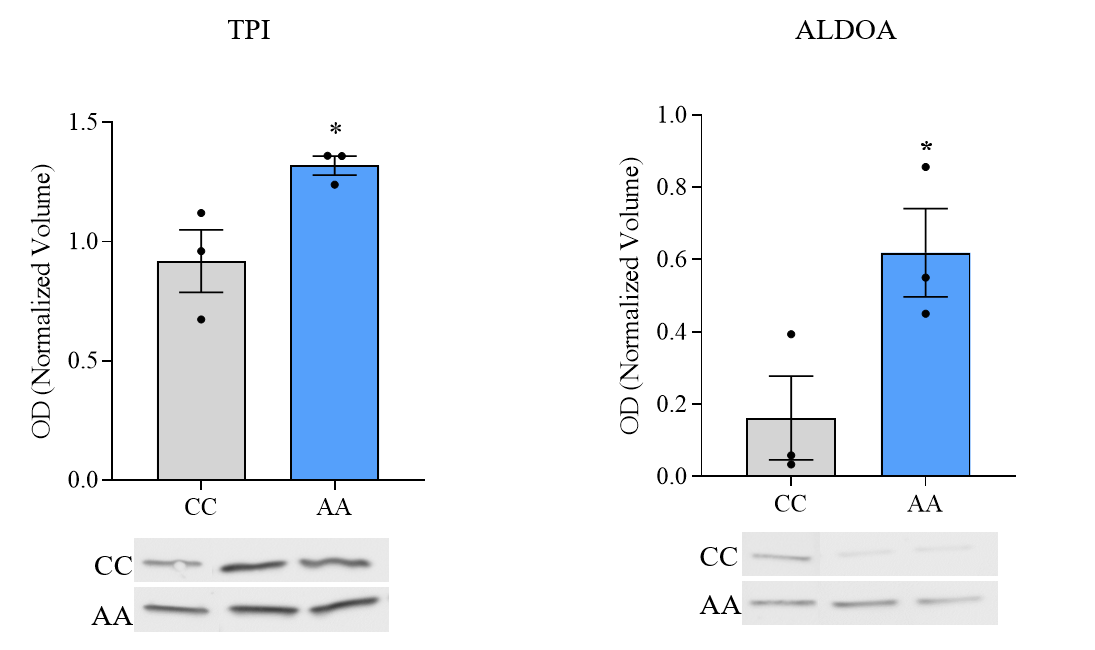

Supplement: Supplementary file 1 [file DataSheet1.ZIP › Supplementary_Matherial/Supplementary_Figure_S3.tif]
